# Ovarian Hyperstimulation Syndrome: Current Views on Pathophysiology, Risk Factors, Prevention, and Management

**Published:** 2009-06-10

**Authors:** Michael M Alper, Laura P Smith, Eric Scott Sills

**Affiliations:** Boston IVF, Waltham MA; a Department of Obstetrics & Gynecology, Division of Reproductive Endocrinology & Infertility, Beth Israel Deaconess Medical Center & Harvard Medical School, Boston, MA; b Division of Reproductive Endocrinology and Infertility, Department of Obstetrics and Gynaecology, School of Medicine, Royal College of Surgeons in Ireland/The Sims Institute, Sims International Fertility Clinic; Dublin, Ireland

**Keywords:** ovarian hyperstimulation syndrome, ascites, coasting, embryo cryopreservation, paracentesis

## Abstract

**Objective:**

To summarize current views on the pathophysiology, risk factors, prevention, clinical features, and management of Ovarian Hyperstimulation Syndrome (OHSS).

**Design:**

Literature review

**Results:**

OHSS is a condition characterized by increased capillary permeability, and experimental evidence has identified a provocative link to pathologic vasoactive cytokine actions. Although the ultimate physiologic mechanism of OHSS is not yet known, there are well-known risk factors that must be considered during the administration of medications to treat infertility. Clinical features are consequences of third-spaced intravascular fluid, and OHSS may become life-threatening secondary to thromboembolism or compromised pulmonary or cardiovascular function. Cornerstones of prevention have historically included cycle cancellation, coasting, decreased dosing of human chorionic gonadotropin (hCG) trigger, use of an agonist trigger, and cryopreservation of all embryos. Newer methods of prevention include the administration of a dopamine agonist medication. Management options for OHSS include outpatient transvaginal paracentesis, outpatient transabdominal paracentesis, and inpatient hospitalization with or without paracentesis.

**Conclusions:**

OHSS continues to be a serious complication of assisted reproductive therapy (ART), with no universally agreed upon best method of prevention. Coasting and cryopreservation of all embryos are the most commonly used approaches in the literature, but cycle cancellation is the only method that can completely prevent the development of OHSS. Dopamine agonists are currently being investigated to both prevent and improve the clinical course in OHSS. Recent publications suggest that outpatient paracentesis both prevents the need for inpatient hospitalization and is a cost-effective strategy.

## Introduction

Ovarian Hyperstimulation Syndrome (OHSS) is a rare, iatrogenic complication of ovarian stimulation with follicle stimulating hormone (FSH) medications. OHSS was first described in 1943, and the first fatal cases were documented in 1951. Severe OHSS is estimated to occur in approximately 1% of all gonadotropin cycles. A study done in 2005 using data from the Finnish registry reported an incidence of severe OHSS of 1.4% per cycle, with an individual risk per patient of 2.3% over a mean number of 3.3 cycles (1). Using population data from European (2003) and United States (2005) in vitro fertilization (IVF) databases, it can be estimated that there were a total of 300,000 IVF cycles reported in Europe and 130,000 IVF cycles reported in the United States, for a total of approximately 430,000 total IVF cycles annually. If the incidence of severe OHSS were 1.4% per cycle in these 430,000 total IVF cycles, this calculation yields approximately 6,020 patients per year predicted to develop severe OHSS. This calculation lends important perspective on the potential impact of this condition. Notably, mortality is rare but several cases have been reported.

## Materials and Methods

Literature review was performed by a single author (MMA) utilizing the following search terms in the PubMed database: ovarian hyperstimulation syndrome, in vitro fertilization, IVF, trigger, GnRH agonist, embryo cryopreservation, albumin, dopamine agonist, and paracentesis.

## Results

### Pathophysiology

The underlying feature of OHSS is increased capillary permeability leading to a fluid shift from the intravascular space to third space compartments. Factors including molecules in the renin-angiotensin system and vascular endothelial growth factor (VEGF) have been implicated in the pathophysiology of OHSS. The renin-angiotensin system is widely known to regulate fluid balance, and was among the first systems investigated as a potential contributor to the findings in OHSS. The presence of an ovarian renin-angiotensin system has been long identified, and Derck et al. in 1987 found follicular fluid (FF) from IVF patients stimulated with human menopausal/human chorionic gonadotropin to have markedly elevated prorenin levels (40x plasma) and renin levels (10x plasma) compared to unstimulated controls (2). Likewise, Lightman et al. identified elevated FF levels of renin, angiotensin II, and angiotensin III from stimulated compared to unstimulated controls (3).

More recently, investigations have shifted to focus on the relationship of VEGF and OHSS. Vascular endothelial growth factor is a vasoactive glycoprotein (cytokine) which stimulates endothelial cell proliferation, cell permeability, and angiogenesis. VEGF mRNA has been found to be expressed in granulosa cell culture. Furthermore, VEGF levels have been shown to increase in response to the administration of luteinizing hormone (LH), follicle stimulating hormone (FSH), and hCG in granulosa cell culture. Levin et al. evaluated the relationship between VEGF and OHSS in an elegant series of experiments (4). They studied FF from 80 women undergoing gonadotropin induction for the treatment of infertility. The FF samples were separated into four groups (I – IV) based on the number of oocytes retrieved. Using an in vitro endothelial cell permeability assay, the authors found that patients producing more eggs had increased capillary permeability. Furthermore, there was a direct correlation between the number of eggs retrieved and the FF VEGF level, with statistically significantly more VEGF in FF from patients who produced more oocytes. They investigated the mechanisms of increased cell permeability using an actin stain of tight junctions in endothelial cells. Treatment with FF and VEGF produced equal redistribution of the actin filaments in the tight junctions, suggesting that VEGF is the FF component responsible for the changes in vascular permeability characteristic of OHSS.

Other possible mechanisms for the development of OHSS have been suggested, including FSH receptor variability. De Leener et al. have investigated FSH receptor mutations and the development of spontaneous OHSS in order to shed further light on spontaneous and iatrogenic induction of OHSS (5).

### Risk Factors

There are many well-known and clearly-documented risk factors for the development of OHSS including: young age, low body mass index (BMI), polycystic ovarian syndrome (PCOS), allergic history, high antral follicle count, high doses of gonadotropins, high or rapidly rising estradiol levels, large numbers of large and medium-sized follicles, large numbers of eggs retrieved, high or repeated doses of hCG, pregnancy, and prior OHSS (6). Of these risk factors, high or rapidly rising estradiol levels are particularly unreliable and over-rated predictors of OHSS (7). An estradiol cut-off of 3,000 pg/mL will miss 2/3 of patients with severe OHSS. Counting the number of 12mm follicles is actually a better predictor of OHSS than serum estradiol levels, and the combination of an estradiol level of 5,000 pg/mL and eighteen 12mm follicles was found to be the best predictor of OHSS in this study, yielding a sensitivity of 83% and specificity of 84% (7). The relative uselessness of serum estradiol level to predict the development of OHSS was confirmed in a study done at our center, in which 183 patients with OHSS requiring hospital admission or outpatient transvaginal paracentesis were evaluated. Of these 183 patients who required an intervention secondary to OHSS, 71% had a peak estradiol level less than 3,000 pg/mL, 12% had a peak estradiol between 3,000 pg/mL and 4,000 pg/mL, and only 17% had a peak estradiol greater than 4,000 pg/mL (Boston IVF data, 1999–2007, unpublished).

### Prevention

Cornerstones of OHSS prevention have historically included the following strategies: cycle cancellation with withholding of hCG trigger, coasting, decreasing the dose of hCG trigger, agonist trigger, and cryopreservation of all embryos. Newer techniques include intravenous (IV) albumin at the time of egg retrieval and the use of dopamine agonists.

IVF cycle cancellation is the most effective preventative technique, but is emotionally and financially stressful for all involved. Cycle cancellation is generally reserved for patients with a history of severe OHSS in a prior cycle and in cases of total loss of control of the cycle.

Coasting involves temporarily stopping gonadotropin administration and postponing the hCG trigger until the estradiol level is lower. The proposed mechanism of coasting is as follows: lower gonadotropin stimulation leads to decreased LH receptors, leading to decreased luteinization, and subsequent decreased VEGF levels. Lower gonadotropin stimulation may also increase the rate of granulosa cell apoptosis, especially of smaller follicles. Coasting lowers the level of follicular fluid VEGF, thereby potentially preventing the development of OHSS (8).

Close evaluation of the literature on coasting and OHSS reveals that the true effectiveness of coasting cannot be determined since there are no randomized, controlled trials investigating the implementation and outcomes of this strategy. The documented indications for coasting are variable among current studies, as is the target estradiol level (typically approximately 3,000 pg/mL). Coasting does not even totally prevent the development of OHSS, and in a study by Delvigne et al., 16% of patients who were coasted still had ascites and 2.5% still required hospitalization (9). Furthermore, coasting for greater than four days has been shown to result in lower pregnancy and implantation rates (10, 11).

The strategy of using a lower dose of hCG trigger is included among options to prevent the development of OHSS. Schmidt et al. in 2004 did a retrospective of high responders, and included 194 IVF cycles (12). In these patients, if the estradiol level ranged from 2,500 pg/mL to 4,000 pg/mL, the patients were given 5,000 IU hCG trigger. If the estradiol level was above 4,000 pg/mL, they were given a lower trigger dose of only 3,300 IU hCG. Despite the decreased dose of hCG trigger administered, there was no difference in OHSS, but there was excellent oocyte maturation even with a dose of hCG of 3,300 IU. A study by Kolibianakis et al. in patients with PCOS showed similar results when patients were administered either an hCG trigger of 10,000 IU, 5,000 IU, or 2,500 IU (13). In these groups, there was no difference in pregnancy rate (29% vs. 30.8% vs. 34.8%) or the development of severe OHSS (3.8% vs. 3.8% vs. 0%) even with an hCG trigger as low as 2,500 IU. Unfortunately, although theoretically it makes sense to reduce the dose of hCG, there is little data to support this practice and studies are either limited by small sample size or not powered to detect a difference.

Using an agonist medication to trigger ovulation has been proposed as another strategy to prevent OHSS. Agonist trigger can only be used in the setting of an antagonist protocol, and Leuprolide 1mg or Triptorelin 0.2mg have been suggested. This method was first described by Itskovitz-Eldor in 2000 in the treatment of eight patients with OHSS (14). Review of the literature identifies 23 articles published on the topic of agonist trigger and prevention of OHSS. A meta-analysis by Griesinger et al. in 2006 identified only 3/23 of these studies which met criteria for meta-analysis (randomized, controlled trials) (15). Comparing pregnancy rate per patient randomized to GnRH agonist vs. hCG trigger identified no difference in number of oocytes retrieved, fertilization rate, or embryo score. No patients randomized to either GnRH agonist or hCG trigger developed OHSS; but there was the suggestion of a lower pregnancy rate in patients who had GnRH agonist trigger, possibly due to a luteal support problem (15).

Cryopreservation of all embryos in patients with OHSS or at risk for developing OHSS is a fifth strategy which has been employed. Sills et al. in 2008 analyzed outcomes in patients undergoing elective embryo cryopreservation to prophylax against the development of OHSS (16). They identified 51/2,892 IVF cycles (1.8%) with patients who were at risk for OHSS (> 20 oocytes retrieved and presence of documented ascites) and had elective cryopreservation of embryos at the 2PN stage. The mean number of oocytes retrieved was 23 and mean number of 2PN embryos frozen was 14. The mean number of days from embryo cryopreservation to date of thaw embryo transfer was 115 (range 30–377), and embryo blastulation rate was 88%. Finally, the live birth rate per embryo transfer was an excellent 43.6% in these patients.

Certainly the concept of cryopreservation of all embryos seems logical as a strategy to prevent OHSS given that OHSS is more common and more severe with pregnancy due to hCG-induced intrinsic ovarian stimulation. However, the Cochrane review found insufficient evidence in the literature to support the routine practice of embryo cryopreservation to prevent OHSS (17). As with all methods, it may reduce but not eliminate OHSS. Other studies evaluating the usefulness of embryo cryopreservation to prevent OHSS have shown either equivalence between cryopreservation and coasting (18) or equivalence between cryopreservation and administration of IV albumin (19), but no overwhelming superiority of embryo cryopreservation.

The administration of IV albumin has been suggested as an alternate method to prophylax against the development of OHSS. Literature review identifies the largest and best study investigating this topic to be a randomized, controlled trial by Bellver et al. in 2003 (20). In this study of 976 patients at high risk for the development of OHSS (20 eggs), the patients were randomized to either the administration of IV albumin 40 gm at the time of vaginal oocyte retrieval or a placebo arm receiving no treatment x 30 minutes. They found no difference in the number of patients requiring paracentesis to treat OHSS, need for hospitalization, or time to resolution of symptoms. They concluded that there was no benefit to giving IV albumin prophylactically, and that the theoretical risks of transmission of prions, viruses, or Creutzfeld-Jacob disease outweighed any theoretical potential benefit.

Finally, the most recently suggested strategy to prevent the development of OHSS is the use of dopamine agonists such as Cabergoline. The proposed mechanism is inhibition of phosphorylation of the VEGF receptor by Cabergoline, thereby preventing increased capillary permeability, the main action of VEGF (21). The dopamine agonist Cabergoline has been investigated in healthy egg donors at risk for OHSS (20 or more oocytes) by Alvarez et al. in 2007 (22). In this study, patients were assigned to receive either Cabergoline 0.5mg/day for 8 days starting on the day of hCG (35 patients) or placebo (32 patients). All patients underwent evaluation for OHSS, including serum hematocrit, ultrasound to evaluate for ascites, and magnetic resonance imaging (MRI) for ovarian venous permeability. They found that in the women who received Cabergoline, the ascites volume was statistically significantly lower than in those who received placebo. They also found that the percentage of women who developed moderate OHSS was statistically significantly lower in those patients who received Cabergoline (7/35 [20%] vs. 14/32 [43.8%]). There was no difference in the percentage of patients in each group who developed severe OHSS.

### Classification and Clinical Features

There have been many different classification systems for OHSS proposed, which generally identify a mild, moderate, and severe subtype with varying internal grades of severity. In order to simplify the classification of OHSS, we use a classification system adapted from that proposed by the Royal College of Obstetricians & Gynaecologists in 2006 (23). We identify mild OHSS, in which patients have abdominal bloating and mild abdominal pain; moderate OHSS, characterized by nausea, vomiting, moderate abdominal pain, and ultrasound evidence of ascites; severe OHSS, identifiable by clinical ascites, oliguria, hematocrit > 45%, and hypoproteinemia; and critical OHSS, with tense ascites, oliguria or anuria, hematocrit > 55%, and white blood count > 25,000.

The clinical features of OHSS are easily identifiable by the above classification strategy and are logical in the context of the underlying pathophysiology of increased capillary permeability. Patients with moderate OHSS complain of lower abdominal pain, abdominal distention, and gastrointestinal symptoms including nausea, vomiting, and diarrhea. Patients classified as having severe OHSS have rapid weight gain, tense ascites, hemodynamic instability, respiratory difficulty, progressive oliguria, and laboratory abnormalities including hematocrit > 48%, hyponatremia (< 135mEq/L), hyperkalemia (>5.0mEq/L), and elevated creatinine (>1.2mg/dL).

The clinical features found in each organ system can also be identified, and patients with OHSS of increasing severity experience increased numbers of affected organ systems (24). Gastrointestinal system findings include ascites (third-spacing of fluid), paralytic ileus, and enlarged ovaries. Pulmonary system findings include pleural effusions, restrictive lung disease from ascites or paralytic ileus, and ARDS. Cardiovascular system findings include decreased intravascular volume, decreased blood pressure, decreased central venous perfusion, and compensatory increased heart rate and cardiac output with arterial vasodilation. Coagulation abnormalities include hemoconcentration, increased estrogen level leading to hypercoagulability, and thrombosis (venous > arterial). Renal system findings include decreased renal perfusion with subsequent oliguria or renal failure. Hepatic system findings may include hepatic edema. Constitutional symptoms occur as well, with 50% of severe cases presenting with elevated temperature of non-infectious etiology. The etiology of hyperthermia is presently unclear, and may be due to cytokines or prostaglandins. Hematologic findings include increased hematocrit (secondary to increased capillary permeability and fluid loss) and elevated white blood cell count, a multifactorial finding associated with elevated estrogen level, prostaglandins, and dilution. Gynecologic findings include enlarged ovaries which may torse or rupture. Finally, electrolyte findings classically include hyponatremia (secondary to increased antidiuretic hormone due to decreased intravascular volume) and hyperkalemia (secondary to the renal sodium/potassium pump alterations).

### Outpatient Management

Paracentesis of abdominal ascites was first described in 1994, with the use of outpatient abdominal paracentesis recommended to prevent hospitalization (25). In 1998, aggressive outpatient single transvaginal paracentesis (and IV albumin) was identified to prevent hospitalization (26). Subsequently, in 2000 repeated transvaginal paracentesis (one to three times) and IV albumin was found to prevent hospitalization (27). In 2008, we performed a retrospective review at our center of all patients diagnosed with OHSS requiring paracentesis, hospitalization, or expectant management (28). Between 1999 and 2007, from a total of 9,707 patients (20,538 IVF cycles), 183 patients were identified with the diagnosis of OHSS (1.8% of patients). In 2002, we began using outpatient transvaginal paracentesis in the management of OHSS. The technique for outpatient transvaginal paracentesis that we employ involves the standard vaginal oocyte retrieval set-up. The tubing is removed from the vaginal oocyte retrieval needle, and the needle hub is attached to standard wall suction tubing set at wall suction pressure of 200mm mercury. The tubing is attached to wall suction, the needle advanced through the vaginal wall into the peritoneal cavity, and as much fluid is removed as can be removed.

In our review of the experience with outpatient transvaginal paracentesis in the management of OHSS, we identified 146 paracenteses performed in 96 patients. The majority of patients underwent a single paracentesis (50/96 patients, 52%), 36% (35/96 patients) required a second, 8% (8/96 patients) underwent a third, 3% (3/96 patients) underwent a fourth, and one patient (1%) required five paracenteses. The mean volume of ascites fluid removed was 2,155mL (range 500 – 4,500cc). We found that with the initiation of outpatient transvaginal paracentesis as the primary management for OHSS in 2002, there was a dramatic decrease in the number of patients requiring hospitalization for OHSS. We identified no complications from paracentesis, and all patients had symptomatic improvement.

A recent cost analysis was published comparing the cost of outpatient management of OHSS with paracentesis versus inpatient management (29). Using mathematical modeling, the authors estimated that conservative inpatient management of OHSS would cost $10,099 compared to $1,954 for outpatient management using paracentesis. Given this dramatic difference in costs, they concluded that early outpatient paracentesis is the most cost-effective management strategy for moderate to severe OHSS.

Clearly, outpatient management of patients with OHSS must be diligently enacted on the part of both the provider and the patient. We instruct patients who are being managed as outpatients on the symptoms of OHSS, with strict instructions to contact their team immediately with any worsening symptoms. They are instructed to avoid excess fluid intake and drink “to thirst”, approximately 1 liter daily. “Sports drinks” can be used if desired. They are given prescriptions for analgesia and antiemetics as needed. Strenuous activity and intercourse are to be avoided, but we advise against strict bedrest. Patients are asked to record daily weight and estimated daily urine output (or number of voids and degree of concentration). Progesterone supplementation is continued in the luteal phase, but supplementation is never done with hCG. In patients diagnosed with moderate or severe OHSS, we check baseline and serial serum laboratory values including: baseline clotting studies, hematocrit, white blood cell count, electrolytes, BUN, creatinine, liver function tests, and protein. We check ultrasound examinations for ascites, ovarian dimensions, and pleural effusions. Chest roentogram is ordered if patients complain of pulmonary difficulties. Echocardiogram and electrocardiograms are ordered if there is suspicion of pericardial effusion. All patients with OHSS being managed as outpatients are examined by a physician, with routine examination always including vital signs (blood pressure, pulse, weight), pulmonary exam, cardiovascular exam, abdominal exam, and waist circumference measurement. A flowsheet with the key symptoms and measurements is given to all patients, along with instructions for form completion.

### Inpatient Management

In a small percentage of patients, hospitalization for OHSS will be required. Indications for hospitalization include social factors such as patients who live far away from medical care. Medical indications for hospitalization include sicker patients who have symptoms that cannot be managed on an outpatient basis. Such situations include severe abdominal pain or peritonitis, severe nausea or emesis, inability to tolerate oral intake, oliguria, pulmonary compromise, hypotension, dizziness, severe electrolyte imbalance, and severe hemoconcentration. In our center, the decision for inpatient hospitalization is made at the doctor’s discretion following clinical evaluation. In our review of the experience with outpatient management of OHSS, we identified that since the introduction of aggressive outpatient transvaginal paracentesis in 2002, the majority of patients with moderate and severe OHSS have been managed as outpatients.

If hospitalization is required for OHSS, however, we closely manage fluid balance, closely follow laboratory results, and do system-focused therapies as needed. Regarding fluid management, it is critical to correct hemoconcentration; thus, we recommend an initial IV fluid bolus of 500–1,000cc normal saline (NS) over the first hour of admission, followed by rehydration at a decreased rate of 30cc/hour using D5NS titrated to urine output. We reduce oral fluid intake to thirst and patient comfort. Albumin (25%) may be needed for intravascular volume repletion, in which case 50 –100gm of IV albumin is infused over 4 hours and repeated at 4–12 hour intervals as needed. Hyperkalemia is treated in the standard manner with Kayexelate, insulin/glucose, sodium bicarbonate, or albuterol as needed. Rarely, diuretics such as Lasix are given, but extreme caution is used in the administration of diuretics to patients with OHSS given their known intravascular volume depletion. Other inpatient management strategies include paracentesis if needed. Thromboembolism prevention is critical given the hypercoagulatory state of OHSS, and we generally recommend both venous support stockings and anticoagulants such as heparin 5,000 U subcutaneously twice daily. Occasionally, patients may need intensive care unit admission for thromboembolic complications, renal failure, or if invasive hemodynamic monitoring is indicated (central venous pressure, pulmonary capillary wedge pressure).

## Discussion

Review of the literature identifies recent research into the pathophysiology of OHSS which has shed light on VEGF and other angiogenic factors as key targets, but definitive findings on the outcomes and benefits of different strategies for the prevention of OHSS continue to be lacking. Given the shortage of clear data on which to base decisions, clinician opinions are divided regarding the best possible method for the prevention of OHSS, as shown in a paper by Delvigne and Rozenberg in 2001 (30). In this study, clinicians were presented with a case of a 25 year old patient with PCOS undergoing IVF with ICSI, a BMI of 23, and multiple allergies undergoing agonist stimulation protocol using 150U HMG/day x 12 days. The case patient had a peak estradiol level of 6,590 pg/mL and had developed 20 follicles on transvaginal ultrasound. When asked the question “what would you do?” 14% of physicians stated that they would cancel the cycle, 8% would proceed with the vaginal oocyte retrieval as usual, and 78% wanted to do one of the preventative measures against OHSS. Of the physicians desiring to pursue a preventative measure, the majority (60%) would have recommended coasting to decrease the estradiol level, 36% would have advised the use of IV albumen, and 33% would have cryopreserved all embryos, even though the data supporting these preventative strategies is borderline at best.

We have found the technique of aggressive outpatient transvaginal paracentesis to manage OHSS in patients with moderate or severe disease to rapidly improve both patient symptoms and laboratory abnormalities. Further, using this strategy has decreased the need for inpatient hospitalization at our center. Given the favorable cost analysis proposed by Csokmay et al., a management algorithm for OHSS that includes frequent outpatient transvaginal paracentesis seems both medically sound and economically advantageous (29).

In summary, OHSS continues to be a serious complication of ART and conclusions are lacking in the literature regarding the best method of prevention. Coasting is the most common method used, followed by cryopreservation of all embryos. Data from randomized, controlled trials is limited for all suggested preventative strategies. Furthermore, it is difficult to prove that one method is superior to another due to the overall low incidence of severe OHSS. Given these limitations, we use the following approach in the prevention of OHSS: (1) coasting when estradiol levels are very high or there are too many medium range follicles; (2) we always give the standard dose of hCG and never cancel the cycle regardless of peak estradiol level; (3) we do not use IV albumin or dopamine agonists; (4) cryopreserve all embryos if the patient is symptomatic on the day of scheduled embryo transfer; and (5) aggressively use transvaginal paracentesis for moderate to severe OHSS.
